# Protein Expression Analyses at the Single Cell Level

**DOI:** 10.3390/molecules190913932

**Published:** 2014-09-05

**Authors:** Masae Ohno, Peter Karagiannis, Yuichi Taniguchi

**Affiliations:** Laboratory for Single Cell Gene Dynamics, Quantitative Biology Center, RIKEN Address, 6-2-3 Furuedai, Suita, Osaka 565-0874, Japan

**Keywords:** single cell analysis, protein expression, stochastic gene expression, burst, single molecule fluorescence microscopy, proteome analysis

## Abstract

The central dogma of molecular biology explains how genetic information is converted into its end product, proteins, which are responsible for the phenotypic state of the cell. Along with the protein type, the phenotypic state depends on the protein copy number. Therefore, quantification of the protein expression in a single cell is critical for quantitative characterization of the phenotypic states. Protein expression is typically a dynamic and stochastic phenomenon that cannot be well described by standard experimental methods. As an alternative, fluorescence imaging is being explored for the study of protein expression, because of its high sensitivity and high throughput. Here we review key recent progresses in fluorescence imaging-based methods and discuss their application to proteome analysis at the single cell level.

## 1. Introduction

Gene expression describes how the genome sequence is decoded into proteins via an intermediate product, mRNA. Despite the relationship between genes, RNA, and proteins, their copy numbers show significant differences. The copy number of the genome in a single cell is constant: at the G1 phase, one haploid has one copy and one diploid has two. In contrast, the copy numbers of mRNAs and proteins fluctuate with time and vary from cell to cell, including even isogenic cell populations, owing to stochastic molecular reactions during gene expressions, such as the stochastic binding of RNA polymerases to the genome and ribosomes to the mRNA [1]. Thus, a single genome sequence can cause a variety of cellular phenotypes by generating different mRNA and protein copy numbers. Although a stochastic mechanism for gene expression may intuitively seem detrimental to cell stability, this property is thought crucial for many biological processes, such as differentiation and evolution [2,3]. One approach to studying this stochasticity and its impact on cell function is single cell gene expression analysis methods.

Because of their numbers, quantifying how many molecules are in a single cell is a tremendous challenge. Single cell transcriptome analysis techniques based on mRNA sequencing and cDNA microarray have made great inroads on this goal [4,5]. Single cell proteome analysis, however, has proven more difficult. Nevertheless, increasing improvements in the sensitivity of techniques such as mass spectroscopy have made this task more achievable [6,7].

Another approach gaining popularity for the characterization of protein expressions in a single cell is fluorescence imaging [1,8,9,10,11,12]. Here, the protein amount can be quantified by imaging proteins that are efficiently labeled with a fluorescent protein. Fluorescence-based methods enable time-lapse measurements [8], because it is not necessary to decompose the observed cell for analysis, which is unlike the case in mass spectroscopy. In addition, fluorescence imaging can measure the localization of the proteins with high resolution at the micrometer level and realize single molecule sensitivity [9,10]. Thus, it offers molecular counting in a single cell with precise quantification of the stochastic gene expression. Currently, the number of protein types that can be imaged at the same time is limited, but several methods for system-wide characterization have been proposed [11,12]. In this review, we highlight recent methods for single cell protein expression analysis based on fluorescence imaging.

## 2. Significance of Single Cell Protein Expression Analyses

For genome-wide analyses in a single-cell, gene expression is routinely determined by measuring mRNA. Recent advances in amplification techniques have greatly aided the detection and quantification of mRNA compared with proteins. Because protein levels are predominantly determined by corresponding mRNA levels, mRNA measuring techniques have been used to estimate protein levels. In fact, this correlation is common across taxa, from bacteria to mammals [13]. 

However, at the same time, several reports have suggested that the protein copy number is determined more by translational control than by transcriptional control, arguing these indirect observations of protein abundance can be misleading. Schwanhäusser *et al.*, calculated the copy numbers and half-lives of mRNA and proteins for over 5000 genes simultaneously in mouse fibroblast cells by pulse labelling followed by mass spectrometry and RNA-sequencing [14]. By combining the measured concentrations and degradation rates of the mRNA and proteins into a mathematical model, they predicted the rates of transcription and translation. The authors concluded that the protein level was controlled more by the translation rate than the transcription rate, demonstrating the importance of measuring not just the transcriptome, but also the proteome when studying cell function. 

**Figure 1:**
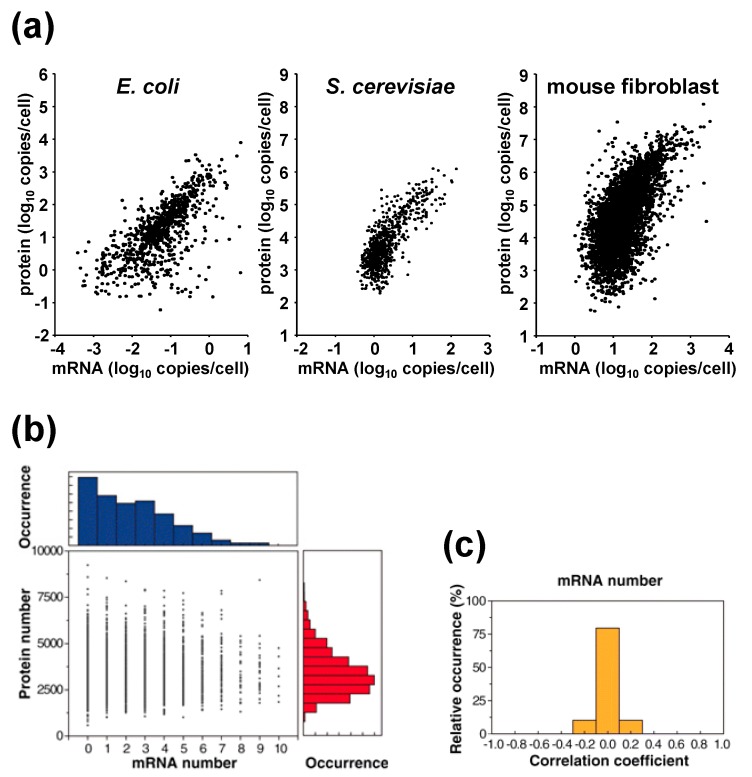
(**a**) Relationship between proteins and mRNA levels across organisms. Data for *E. coli*, *S. cerevisiae*, and mouse fibroblast were adapted from references 12, 15, and 14, respectively. (**b**) Correlation plot of mRNA and protein levels of a TufA gene tagged with YFP in single *E.* *coli* cells. (**c**) Among the 129 genes that were high copy and analyzed, no correlation was observed between mRNA and protein levels in a single *E. coli* cell. Data taken from [12].

Accumulating data also suggest that protein concentrations generally cover a higher dynamic range than mRNA concentrations across organisms (Figure 1a). For example, in *E. coli*, protein copy numbers range from 10^−1^ to 10^4^ per cell, while mRNA copy numbers range far less, from 10^−3^ to 10^1^ per cell [12]. In baker’s yeast, cells were seen to contain 10^1^ to 10^5^ copies of protein, but approximately only 10^−1^ to 10^2^ copies of mRNA [15]. Similarly, in mouse fibroblasts, the copy numbers of protein and mRNA were measured as 10^2^ to 10^8^ and 1 to 10^4^, respectively [14]. The difference in the dynamic range of protein and mRNA copy numbers indicates there exists an amplification step post-transcription. 

Those studies compared protein and mRNA abundances from the same gene in the same species. Others have examined orthologs across diverse species. Comparisons of nematode and fly showed that the protein abundances of orthologous genes correlate better than do the protein abundance and mRNA abundance within the same organism or the mRNA abundances across organisms [16]. Similar results have been obtained by comparing the protein and mRNA abundances of orthologs across seven organisms that range from bacteria to human [17]. In addition, a recent report indicated that the protein levels in the lymphoblastoid cells of three different primates (human, chimpanzee, and rhesus macaque) are more conserved than are the mRNA levels [18]. That report further suggested that protein expression levels evolve under greater constraints than respective mRNA levels. 

The above studies measured mRNA and protein abundances using bulk measurements. On the other hand, Taniguchi *et al.* quantified the abundances simultaneously in a single-cell. They conducted fluorescence *in situ* hybridization (to detect mRNA) in combination with single molecule fluorescence microscopy to measure an *E. coli* fluorescent-protein tagged protein library. They measured 129 highly expressed genes (an example of a correlation plot is shown in Figure 1b) and showed that protein and mRNA copy numbers are uncorrelated at the single-cell level (Figure 1c) [12]. The absence of correlation between protein and mRNA copy numbers in a single-cell can be explained by the different lifetimes of the proteins and mRNAs, as bacterial mRNAs are short-lived (0.6–36 min), whereas their proteins have a long lifetime exceeding cell division (~150 min). Therefore, the abundances of mRNA can only reflect gene expression states over a short period of no more than half an hour, while that of proteins can reflect the accumulated results of gene expression for a period that goes beyond the duration of the cell cycle. This result offers a cautionary note for single-cell transcriptome analysis and suggests the importance of single cell proteome analysis.

## 3. Stochastic Nature in Gene Expression

Stochasticity in gene expression is often dominated by “bursting”, a phenomenon that describes how multiple gene expression products are generated during intermittent active periods that are separated by inactive periods. According to the central dogma, two types of bursts exist, transcriptional bursts and translational bursts. Regarding transcriptional bursts, many mRNA molecules are produced in a short period rather than with a constant probability per unit time. On the other hand, in translational bursts, many protein molecules are produced from a single mRNA when the rate of protein translation is greater than that of mRNA degradation.

**Figure 2:**
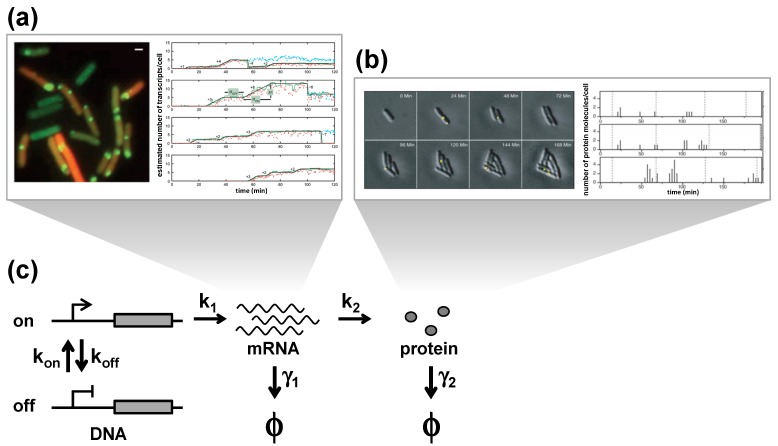
Stochastic gene expression. (**a**) Experimental observations of transcriptional bursts. Expressions of mRNA molecules in single *E. coli* cells are visualized in real-time by labeling the MS2-GFP system [19]. Each green spot consists of one or more mRNA molecules (**left**). Time trace of mRNA expression in single cells (**right**). (**b**) Experimental observations of translational bursts. The expression of proteins labeled with Venus in single *E. coli* cells are visualized in real-time. Venus molecules are photobleached immediately after the image acquisition so that only newly synthesized proteins are imaged each time [10]. Each yellow spot represents single protein molecules (**top**). Corresponding time courses (**bottom**). (**c**) Model for stochastic gene expression. The promoter transitions between an inactive ‘off’ state and an active “on” state with rates k_on_ and k_off_. During the on state, mRNAs are transcribed at the rate of k_1_, and proteins are translated at the rate k_2_. mRNAs and proteins are degraded at the rates γ_1_ and γ_2_, respectively. The states of degraded mRNAs and proteins are indicated as ϕ. Images are taken from [10,19].

Experimentally, transcriptional bursts have been observed in single *E. coli* cells (Figure 2a) [19,20], eukaryotes [21], and other higher eukaryotes [22]. Real-time observation of mRNA expression in living cells has demonstrated that genes are transcribed in a discontinuous fashion. To visualize mRNA molecules, the MS2 system has been used [19,20,21,22]. This approach exploits the specific interactions between the MS2 hairpin RNA and a complex that includes a fluorescent protein and the MS2 capsid protein. Additionally, luminescence imaging has revealed the transcription kinetics of the short-lived luciferase reporter system [23], and RNA FISH experiments allow us to analyze variations in the number of mRNA molecules that result from transcriptional bursts without the use of a genetic tag [24,25,26,27]. 

There are several possible mechanisms that explain the transcription burst phenomena [28]. One is promoter on/off switching. The binding and unbinding of regulatory molecules, such as histones and transcription factors, may also be a cause. A recent *in vitro* assay has indicated that DNA supercoiling in the promoter region may also be responsible [29]. In addition, global factors that affect gene activities at the whole gene level, such as cell-cycle-dependent regulation of the promoter activity and the cooperative recruitment of RNA polymerases, are also considered possible causes [28].

Similarly, the observation of translational bursts has been reported. One study monitored the production of single proteins in single *E. coli* cells by imaging newly synthesized fluorescent-fused proteins under the control of the lac promoter (Figure 2b) [10]. The same study demonstrated that proteins were generated in bursts from a single mRNA, with each burst generating an average of 4.2 proteins. The same lab acquired similar results when applying the enzymatic amplification method on the same lac promoter and another reporter gene, β-galactosidase, in *S. cerevisiae* [9]. 

Bursting behaviors can be described with a simple kinetic model, where transcription, translation, promoter switching, and the degradation of mRNA and proteins, are assumed as first-order reactions (Figure 2c). This model was first introduced by Peccoud and Ycart [30] and is now broadly used to understand quantitative gene expression at the single molecule level. The rates of transition between the on and off states are indicated in the figure as k_on_ and k_off_, respectively. The rate of transcription in the on state is described as k_1_, and the rate of translation as k_2_. The rates of degradation for mRNA and protein are defined as γ_1_ and γ_2_, respectively. Transcriptional bursting occurs during the on state. The number of mRNAs produced in a single transcriptional burst, or transcriptional burst size, is given by k_1_/k_off_. The transcriptional burst frequency changes with the duration of the inactive period (k_on_). In contrast, the translational burst size, which denotes the number of proteins per single mRNA, is given by k_2_/γ_1_. This means that bursts of protein expression occur when k_2_ is greater than γ_1_, which is the case for most genes in budding yeast [31].

Because γ_1_ is much greater than γ_2_, the steady-state distribution of mRNA and protein copy numbers among a cell population can be given by solving the master equations for this model [31]. The probability density function of having m mRNAs at steady-state is:

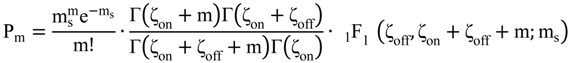

where m_s_ = k_1_/γ_1_, ξ_on_ = k_on_/γ_1_, and ξ_off_ = k_off_/γ_1_. _1_F_1_(a,b;z) is the confluent hypergeometric function of the first kind. In contrast, the probability of having n proteins is given by:

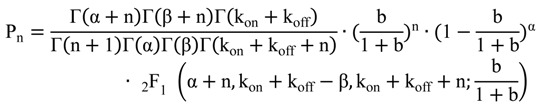

where a = k_1_/γ_2_, b = k_2_/γ_1_, α = (1/2)(a + k_on_ + k_off_ + ϕ), β = (1/2)(a + k_on_ + k_off_ − ϕ) and ϕ = (a + k_on_ + k_off_)^2^ − 4ak_on_. _2_F_1_(a,b,c;z) is the Gaussian hypergeometric function. Assuming that DNA is always active at steady state (k_off_ → 0), this equation becomes a negative binomial distribution [32]. Moreover, assuming a continuous variable for n leads to a gamma distribution [33], which has been used to analyze the distributions of protein concentrations (but not protein copy numbers) in single *E.* *coli* cells, and minimizes any effects on the analysis caused by abrupt changes in protein numbers before and after cell division [12]. In fact, it has been shown that in *E. coli* the distribution of almost all protein concentrations can be described by a gamma distribution.

In the above equation, the protein noise, defined by η^2^ = <n^2^>/<n>^2^, satisfies:

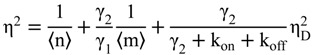

where <m> and <n> are the mean numbers of mRNA and protein, respectively, and η_D_^2^ is the noise when DNA is in its active state and equals k_off_/k_on_. This equation demonstrates that the noise in protein expression levels can be influenced by any rates of translation, transcription or promoter switching, which is consistent with an expanded model [34].

The above model attributes intrinsic noise in protein copy numbers to the stochasticity inherent in gene expression. Additionally, a previous work has shown the existence of another noise source, extrinsic noise, due to fluctuations in other cellular components [1]. Subsequent work has indicated that extrinsic noise is inherited over a timescale of about one cell cycle, while intrinsic noise decays rapidly [8]. Extrinsic noise can be modeled in the above simple kinetic model by adding static or slowly varying heterogeneities in individual rate constants among cells [12]. In the *E. coli* case where the intrinsic noise distribution is described as a gamma distribution with two adjustable parameters, P_n_(a_E_, b_E_), the total noise distribution can be expressed as:

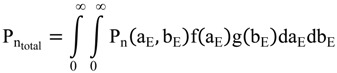

where it is assumed that a_E_ and b_E_ exist with distributions of f(a_E_) and g(b_E_), respectively. Thus, the noise, η_total_^2^, is given by:

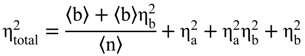

where η_a_^2^ = <a_E_^2^>/<a_E_>^2^ and η_b_^2^ = <b_E_^2^>/<b_E_>^2^. The extrinsic noise in the last three terms predicts the existence of an unavoidable, global noise floor independent of the mean protein abundance, <n>, which is consistent with previously reported experimental observations [12].

Taken together, adding to the simple kinetic model described in Figure 2 the assumption of static heterogeneities in kinetic rate constants among cells can describe both intrinsic and extrinsic noise at the single cell level. These mathematical descriptions quantitatively connect the steady-state distributions of mRNA and protein copy numbers among cells with fluctuations of mRNA and protein copy numbers over time within a cell lineage. More importantly, the models are conducive for understanding the complexity of gene expression behaviors at the single cell level.

## 4. Single Molecule Detection of Protein Expression

As noted above, fluorescence imaging can provide quantitative measurements of protein expression levels in single cells, which are essential to understanding cell dynamics and cell heterogeneity. One common approach to quantifying protein copy numbers in a single living cell is the use of a genetic tag. Typically, the coding sequence of a fluorescent protein is genetically inserted downstream of a promoter or is fused to or replaced with an open reading frame of the protein of interest so that the target promoter or protein expression coincides with the expression of the fluorescent protein. Protein expression levels can thus be monitored in real-time with fluorescence microscopy by evaluating integrated intensities within the cells. An alternative way to study protein expression levels is to label proteins with fluorescence antibodies in fixed cells. This method, unfortunately, does not perform real-time monitoring of protein levels and is highly affected by the recognition ability of the antibody. However, it also has no need for *a priori* genetic manipulation and causes no artifactual perturbation in the genetic tagging, which makes it suitable for many purposes in both scientific research and clinical diagnosis. 

Concentrated efforts on new technologies have led to the detection of single molecules in single cells by single-molecule fluorescence microscopy. Such sensitivity promises new insights on gene expression, including the ability to detect the copy numbers of proteins, which can then be used to characterize protein localization and diffusion with high spatiotemporal resolution. For example, Choi *et al.*, monitored stochastic phenotype switching in the carbohydrate metabolism of single *E. coli* cells [35]. Below we introduce several methods for single molecule detection.

**Figure 3:**
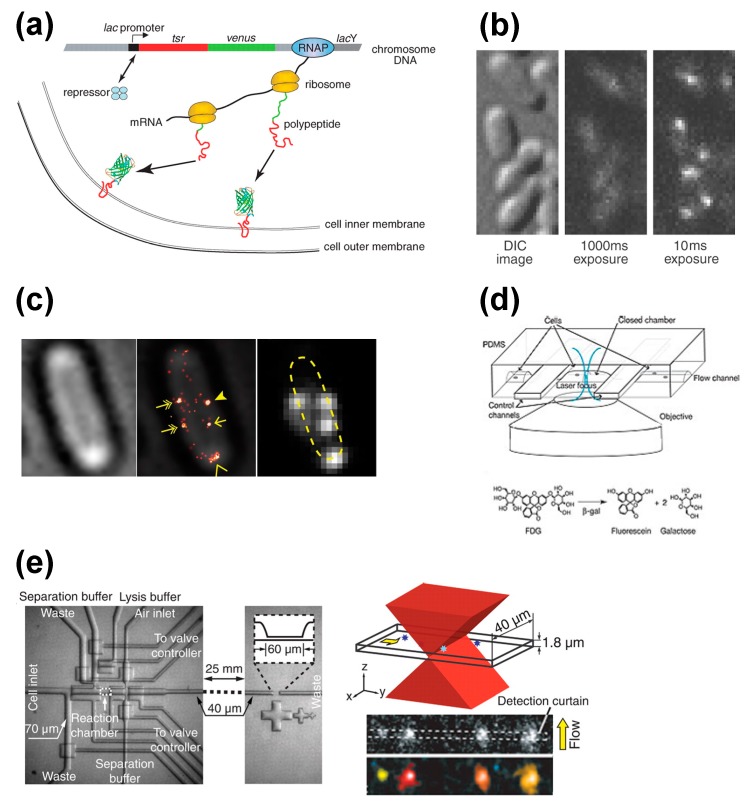
Selected methods for single cell protein expression analysis. (**a**) Single molecule detection of protein expression at the membrane. The inclusion of a membrane protein (Tsr) into the gene assures the protein of interest will be localized at the membrane, while the inclusion of a fluorescent protein (Venus) into the gene assures detection of the membrane localization [10]. (**b**) Stroboscopic detection of single fluorescent proteins in single *E. coli* cells [37]. (**c**) Counting the number of FliM-Dendra2 in a *E. coli* cell with PALM/STORM. Bright-field image, super-resolution Flim-Dendra2 overlay image and diffraction-limited Flim-Dendra2 image are shown [41]. (**d**) Microfluidic system for monitoring single reporter molecules with the enzymatic assay. Schematic diagram of fluorogenic enzymatic reactions is shown on the bottom [9]. (**e**) Single-cell analysis chip. Schematic diagram of the molecule detection section illuminated by a focused excitation laser is shown on the top right. Examples of recorded frames are shown on the bottom right [43]. Images are taken from [9,10,37,41] and [43].

Yu *et al.*, visualized gene expressions of single living *E. coli* cells at the single-molecule level (Figure 3a) [10], finding they occur in bursts. To investigate the expression of the lac promoter, a complex that includes the membrane protein Tsr and YFP-variant fluorescence protein Venus [36] was inserted downstream. Tsr was used to localize the fluorescence probe to the membrane, as otherwise the complex could diffuse into the cytoplasm leaving its signal to be overwhelmed by the autofluorescence of the cell. Venus was included for several reasons. First, the maturation time of Venus to emit fluorescence (~7 min) is much faster than the cell division time (about 150 min in typical *E. coli* cells in minimal media). Second, the brightness of Venus is sufficient to detect single molecules. Third, the relatively higher wavelength of excitation for Venus (514 nm, *cf.* 488 nm for GFP) can minimize photo-excitation caused by cellular autofluorescence.

However, the inclusion of Tsr or any other membrane protein is a problem if one is interested in protein localization. As an alternative, Elf *et al.*, have proposed a stroboscopic method (Figure 3b) [37]. This method uses short laser pulses (~1 ms) to excite the Venus probe. Because of the short lifetime of the pulse, even diffusing fluorescent protein molecules can be visualized as spots because the diffusion is nearly within the diffraction limit of light. Using this method, they characterized the diffusion states of the lac repressor interacting with the lac operator in living *E. coli* cells.

When quantifying gene expression noise, *i.e*., measuring the distributions of protein copy numbers in a cell population, the above techniques are suitable only when investigating low copy numbers. For high copy numbers, a different strategy should be considered, because the many resulting fluorescent spots are likely to overlap with one another, which compromises the measurement. Taniguchi *et al.*, designed a technique that quantifies gene expression noise for any protein abundance [12]. In this method, distributions of the number of fluorescent probes can be analyzed by deconvolving the measured fluorescence from the cell autofluorescence.

However, even that approach does not allow for the investigation of high protein abundance at the single cell level. Recently, methods based on superresolution microscopy, such as photoactivated localization microscopy (PALM) [38] or stochastic optical reconstruction microscopy (STORM) [39], have been proposed for this purpose (Figure 3c) [40,41,42]. Here, the protein of interest is genetically tagged with a photo-activatable protein such as Dendra2 or mMaple3. The advantage of photo-activatable proteins is that they can be activated incrementally under a weak photoactivation condition, which allows them to be counted individually. One drawback of superresolution microscopy, however, is that it is not suitable for living cell analysis because of the relatively high risk of photodamage due to the long acquisition time. Still, it is arguably the most effective technique for counting proteins in individual cells. 

Fluorescence imaging can be used to study copy numbers without the use of exogenous fluorescence proteins as well, which reduces the risk of both phototoxicity and autofluorescence. Cai *et al.*, monitored protein expressions in single living cells using a microfluidic device and the β-galactosidase assay (Figure 3d) [9]. In this assay, a coding sequence of β-galactosidase is genetically inserted into the gene of interest. While β-galactosidase itself is not fluorescent, it hydrolyzes fluorogenic substrates to produce a fluorescent product. To monitor protein expressions in a single cell, single cells are trapped inside microfluidic chambers and their fluorescence levels are measured. 

Huang *et al.* developed a method for protein counting that needs no genetic tagging nor editing (Figure 3e) [43]. This method uses a microfluidic device that can capture a single cell, lyse it, label the proteins with fluorescently-labeled antibodies, and separate the cell content by electrophoresis. The protein number can be determined by counting the number of molecules during migration. The group measured the β_2_ adrenergic receptor with high counting efficiency, finding this protein can vary from just a few thousand to several tens of thousands in SF9 insect cells. Although this approach cannot analyze the dynamics of gene expression, it is well suited for diagnostic purposes.

## 5. Platforms for System-Wide Characterization

Because any given cell function can be controlled by the expression of thousands of genes, systems-wide characterization is needed. Such a task is not trivial for fluorescence-based methods, because the broad excitation/emission spectrum seen for many fluorescent probes limits the number of probe species that can be analyzed independently and consequently the number of molecule types that can be analyzed. For mRNA analysis, Levsky *et al.* offered a solution by proposing combinatorial labeling, which labels each mRNA species with different combinations of several different dyes [44]. This barcoding strategy increases the number of unique fluorescence spectra, and is especially advantageous when using superresolution microscopy to discriminate individual mRNA molecules [45,46]. However, applying this strategy to protein analysis is much more challenging, because the copy number of most proteins is much higher and the proteins themselves are harder to discriminate due to them localizing at higher densities.

**Figure 4:**
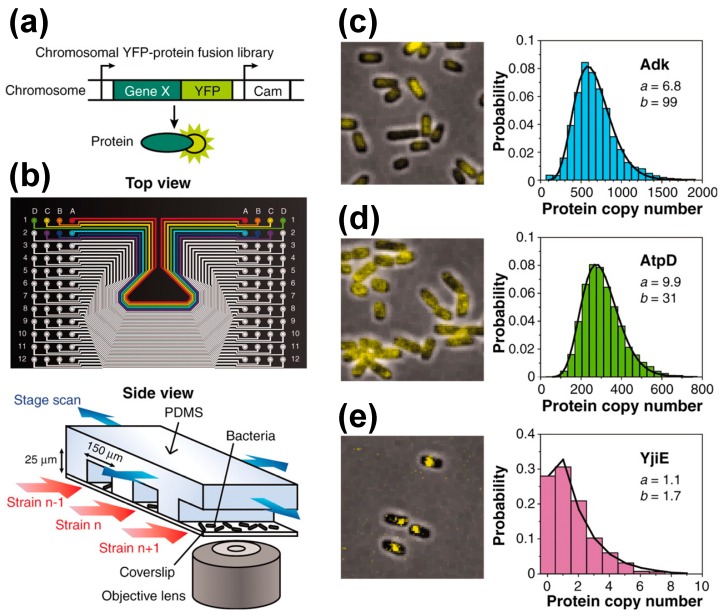
High-throughput single molecule fluorescence microscopy. (**a**) Chromosomal YFP fusion *E. coli* library. In each library strain, a YFP is translationally fused to the C-terminus of a protein in its native chromosomal position. (**b**) Microfluidic chip for imaging 96 library strains. Each strain is immobilized on a polylysine-coated coverslip and is scanned with a single molecule fluorescence microscope. (**c**–**e**) Three examples from a library of *E. coli* strains are shown. Images to the left are representative fluorescent images overlaid on phase-contrast images; histograms to the right are the distribution of copy numbers. Lines are curve fits to gamma distributions with parameters *a* and *b*. Taken from [12].

One standard strategy for system-wide quantification of protein expression levels based on fluorescence imaging is to use a cell strain library. A library is typically a collection of many recombinant cell strains, in which a fluorescent protein is chromosomally fused to different genes [47,48,49]. The cell strains are measured using a high-throughput system from which system-wide information can be acquired. This method cannot measure the proteome in the same single cell, but can take advantages of fluorescence microscopy, which means it can be used to analyze dynamics with single molecule sensitivity. Such cell strain libraries have been constructed for many organisms, including *E. coli* [12], *S. cerevisiae* [47,50], *C. elegans* [48], and human H1299 lung carcinoma cells [51]. Furthermore, correlation analysis of all gene pairs in the entire gene set can describe the entire gene network in a single cell. 

To perform high-throughput analysis of many cell strains, high-throughput flow cytometry is preferred, as this technology automates the delivery of cell strains to a flow cytometer via an autosampling device [11,52]. With this system, approximately seven strains at >50,000 cells per strain can be measured per minute. Newman *et al.* have successfully measured >2500 GFP-tagged yeast strains at single-cell resolution [11]. Unfortunately, the flow cytometry method is inferior in sensitivity and resolution compared to standard cellular imaging. An alternative approach is to use an automated microscope system. The system described above by Taniguchi *et al.* can measure about two strains per minute and ~4000 *E. coli* cells per strain [12] (Figure 4). Accordingly, it was used to measure, 1018 Venus-tagged *E. coli* strains at single-cell resolution and single molecule sensitivity. 

A protein chip approach that does not need any gene recombination has also been proposed [53]. Shi *et al*. developed the single-cell barcode chip, which is a microfluidics system in which single cells are captured and lysed in 2 nL chambers and the released proteins are assayed using an array of antibodies patterned inside the chambers. With this chip, 11 different protein species associated with the phosphoinositide 3-kinase signaling pathway were quantified.

## 6. Future Perspectives

Single live cell analysis with genetic tagging provides information that cannot be acquired by studying ensemble cell populations. The ability to characterize the dynamics and heterogeneity of gene expression will be instrumental in correlating stochastic gene expressions with biological phenomena, as has already been seen in bacterial persisters [54] and the translocation of transcription factors [55]. Similarly, correlation analysis between a large number of genes in single cells is expected to illuminate the network pathways and modules of gene regulations [56]. 

One critical obstacle in live cell analysis is the creation of chromosomal fusion cell strains, especially in higher eukaryote and mammalian cells that have low efficiency of homologous recombination. However, progress is already being made, as random integration via a retrovirus was used to create a chromosomal fusion strain library of human lung carcinoma cells [57]. For efficient genetic engineering, technologies that use site-specific engineered nucleases such as truncated transcription activator-like effector nucleases and clustered regularly interspaced short palindromic repeat RNA-guided Cas9 nuclease have been investigated [58]. It is expected that these technologies will accelerate the generation of complete sets of chromosomal fusion strains in mouse and human cells.

Another obstacle is imaging thicker cells or tissues (*E. coli*, *cf.* 0.5 μm; yeast, 5 μm; mammals >10 μm). In these cases, 3D stacking of images is done using different focal positions. To reduce the strong out-of-focus background that prevents quantitative analyses, 3D sectioning of stacked images is recommended. Confocal microscopy may be one option for this purpose, but it has relatively low sensitivity and the out-of-focus exposure of the excitation laser causes higher photodamage to the sample. In this regard, sheet illumination microscopy may be preferred [59]. This method combines light-sheet illumination with orthogonal camera-based detection to achieve selective plane illumination that minimizes the photodamage. Using similar systems, the imaging of single molecule fluorophores has been achieved in living mammalian cells and tissues [60,61]. 

Meanwhile, developments in single-cell analytic methods not requiring genetic tags will greatly advance diagnostic and clinical work. To analyze protein levels and localizations with fluorescence imaging, fluorescent antibodies are commonly used to label the proteins because of their high specificity. Protein interactions in single cells can also be probed using fluorescent antibodies. For example, multiplex proximity ligation assay was used to analyze the co-existence of two proteins bound to different antibodies that were conjugated with unique tag sequences and rolling circle amplification [62]. These single-cell technologies can visualize protein molecules and complexes at a single-cell level in tissue sections and are sufficiently sensitive for clinical samples. 

Alternatively, mass spectroscopy may not be as sensitive as fluorescent-dependent systems, but it offers to simultaneously measure vast kinds of molecules with high spectral resolution. Recently, single-cell mass cytometry has been reported [63]. Here, cells labeled with antibodies coupled to transition element isotopes are nebulized into single cell droplets, and the metal tags are quantified by time-of-flight mass spectrometry. This method has been used to characterize human born marrow cells with as many as 34 parameters, which is far more parameters than other systems have reported [64]. The high sensitivity is especially advantageous when only having access to a minimal amount of samples from patients, such as biopsies and blood tests. These technologies are expected to reveal differences in phenotypic states of single cells.

Considering the exponential progress in single cell proteomics over the last decade, it is easy to anticipate new innovations that will make currently impractical experiments feasible in the near future. This progress will result in large datasets that are built from single-cell resolution and single-molecule sensitivity imaging on a systems-wide scale. These datasets will present new challenges, as investigators will need appropriate tools to analyze their content. The accumulating information from these techniques is allowing scientists to reveal more and more about the mechanisms and general rules of single-cell gene expressions, which are the basis for heterogeneous single cell activities and complex biological behaviors.
